# Safety principles of lowering inspiratory pressure

**DOI:** 10.1007/s44470-025-00029-9

**Published:** 2026-03-19

**Authors:** Ludovico Messineo, William H Noah, David P White

**Affiliations:** 1 Division of Sleep and Circadian Disorders, Departments of Medicine and Neurology, Brigham & Women’s Hospital & Harvard Medical School, 221 Longwood Avenue, Boston, MA USA; 2 Sleep Centers of Middle Tennessee, Murfreesboro, TN USA

Expiratory pressure relief algorithms (EPRAs) are device features that transiently reduce pressure during expiration with the goal of enhancing comfort. Introduced in the early 2000s [1] to mitigate discomfort associated with exhaling against a positive pressure, they have since become standard in most modern devices. Today, the vast majority of prescribed continuous positive airway pressure (CPAP) devices operate with EPRA enabled [2]. However, several works [3–5], including our novel meta-analysis, have shown that EPRAs do not systematically increase adherence compared to CPAP. Rather, some evidence suggests they may lead to decreased upper airway patency [6]. However, in our meta-analysis, we did not observe a deleterious effect of EPRAs on OSA severity, even though the AHI was slightly greater on EPRAs vs. CPAP (mean difference [95% CI]: +0.33 [−0.05, 0.70] events/h, *P* = 0.09, *I*^*2*^ = 0%, *N* = 774 patients). Although such an increase in AHI was not clinically meaningful, these data suggest that EPRAs might nonetheless increase upper airway resistance, which could partially offset their intended comfort benefits and warrant caution when used in patients with elevated airway collapsibility [7]. It is also possible that, when auto-adjusting devices are utilized, this potentially increased airflow resistance might be sensed by the machine, leading to an increase in therapeutic pressure. This is potentially problematic as higher pressures are often associated with treatment intolerance and poor adherence [8]. Nonetheless, given that real-world harm from EPRAs has not been demonstrated, it remains possible that their benefits and drawbacks vary across patient groups—e.g., individuals with intolerance to expiratory pressure may derive greater benefit—or across diverse, continuously-evolving algorithms (Fig. 1).

**Fig. 1:**
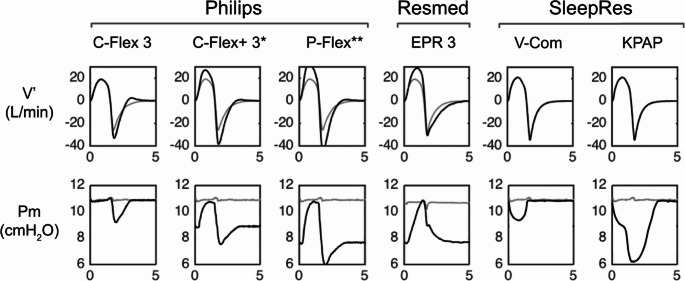
Mask airflow and pressure waveforms of fixed continuous positive airway pressure (CPAP) with (black) and without (gray) pressure relief features from different manufacturers. Waveforms for Philips and ResMed devices were obtained via bench testing, whereas those for SleepRes were drawn for illustrative purposes. C-Flex, at level 3, produces a flow-dependent 3 cmH_2_O drop in expiratory pressure and returns to therapeutic pressure slightly before or at end-expiration. With C-Flex+, pressure decreases by 1 (if therapeutic pressure is 5 cmH_2_O) or 2 cmH_2_O at the transition with expiration, and then a 1–3 cmH_2_O of C-Flex during early expiration is applied. Around end-expiration, C-Flex “wears off”, but pressure remains 1 or 2 cmH_2_O below therapeutic levels (it returns to therapeutic pressure during inspiration). P-Flex operates similarly to C-Flex+, but with a larger pressure drop at the transition with expiration. EPR, at level 3, decreases expiratory pressure by 3 cmH_2_O below therapeutic pressure and maintains this reduction until the next inspiration. V-Com (an inspiratory pressure relief device) provides a flow-dependent drop in inspiratory pressure only (⁓1.5 cmH_2_O at a baseline pressure of 10 cmH_2_O). Kairos PAP (KPAP) delivers a flow-dependent drop (1–3 cmH_2_O) at the start of inspiration, followed by a second 1–3 cmH_2_O drop at peak inspiratory flow, returning to therapeutic pressure after peak-expiratory flow. The combined inspiratory drops cannot exceed 5 cmH_2_O or reduce IPAP below 5 cmH_2_O. Of note, only C-flex, V-Com and KPAP preserve therapeutic pressure at end-expiration, the phase of breathing most vulnerable to pharyngeal collapse. *C-Flex+: identical to A-Flex in APAP mode. **P-Flex: only available in APAP mode *Pm*, mask pressure; *V*’, airflow. The first four panels in each row are reproduced with permission from Zhu et al.^6^. The first two top headings from the left were adapted from Zhu et al., while the subsequent heading and the additional two panels in each row were created by the authors

Subsequent attempts at respiratory cycle-based PAP manipulation focused on reducing inspiratory pressure below expiratory pressure. Thus, inspiratory pressure relief devices [9–12] were created. These consist of a passive resistor inserted into the CPAP circuit, yielding flow-dependent inspiratory pressure reductions (⁓1.5-2 cmH_2_O). Thereafter, an automated device based on similar principles, namely reducing pressure during inspiration, but also maintaining it at a lower level through the first half of expiration, was developed (Kairos PAP or KPAP) [13, 14]. Some have questioned this approach, on the grounds that upper airway collapse is viewed primarily as an inspiratory phenomenon [15]. However, research suggests that the upper airway is most susceptible to collapse at end-expiration (see below). Therefore, it is plausible that inspiratory pressure relief devices and KPAP support the pharynx when it is most needed.

Evidence for pharyngeal airway vulnerability at end-expiration comes from studies using computed tomography (CT) reconstructions or direct observation of the upper airway (fiber-optic endoscopy). The first study with fiberoptic examination of the pharynx in sleeping subjects dates back almost 50 years and observed that upper airway obstruction occurs at end-expiration, and that, at the onset of inspiration, pharyngeal closure is already nearly complete [16]. In three subsequent studies in awake obstructive sleep apnea (OSA) participants and healthy controls [17–19], CT-measured pharyngeal cross-sectional area nadired at end-expiration. By contrast, during early-inspiration (mildly) and early-expiration (more evidently), the upper airway increased in size (Fig. 2). This is especially true in patients with OSA, where larger within-breath changes in cross-sectional area were observed vs. healthy controls [17]. Therefore, it is plausible that the upper airway of OSA patients is more compliant, namely more prone to distend—but also collapse—especially at end-expiration [20], a finding that is also evident in animal models [21]. This is likely due to the fact that pharyngeal dilator muscles activate in sync with inspiration to offset airway negative pressure, which would tend to collapse the airway, and relax during expiration, leaving the pharynx more exposed to the compressive effect of surrounding tissue pressure (e.g., from fat, edema, increased central venous pressure). All this leads to a progressive reduction in cross-sectional area past peak-expiratory flow.

**Fig. 2:**
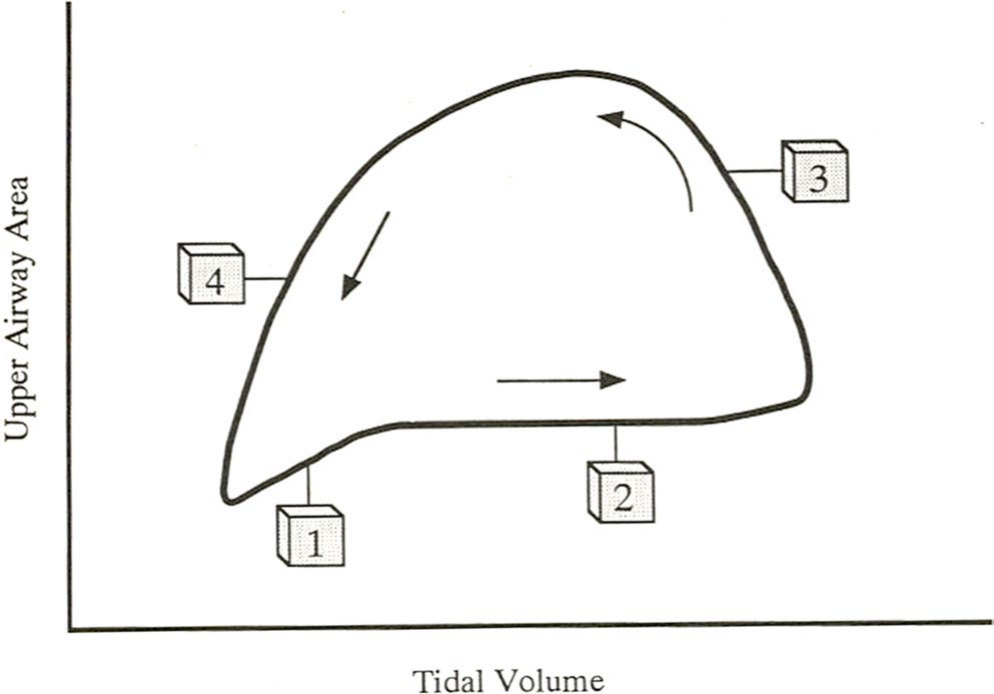
Schematic representation of within-breath variations in cross-sectional area as a function of tidal volume in a participant with obstructive sleep apnea (OSA) during wakefulness. In 1, early inspiration, cross-sectional area increases from its nadir instead of narrowing further, likely due to the action of pharyngeal muscles; In 2, late inspiration, the area remains stable before enlarging in 3 (early expiration), due to positive expiratory pressure in the airway’s lumen. In 4, late expiration, cross-sectional area falls, a pattern more typical of OSA participants. Note the nadir in both cross-sectional area and tidal volume at end-expiration. Breathing follows the direction indicated by the arrows. Reproduced with permission from Schwab et al. [17]

The above findings were confirmed in a larger sample of OSA participants with the use of a fiberscope. In these studies, within-breath variations in cross-sectional area in the breaths immediately preceding respiratory events [22] and in overall stable non-rapid-eye movement sleep [23] mirrored the pattern described in wakefulness. Briefly, early expiration was associated with the largest cross-sectional area, while end-expiration with the smallest. Notably, the possibility of expiratory collapse was also raised by a study demonstrating a progressive increase in expiratory resistance during sleep in general and particularly during the breaths preceding respiratory events in OSA participants [24]. In addition, the expiratory pharyngeal narrowing observed during central hypopneas suggests that many obstructive events may appear central on polysomnography, while in fact occurring due to reduced cross-sectional area in expiration [25].

In order to maintain airway patency during sleep in OSA patients, these decrements in cross-sectional area during late expiration should be addressed primarily with positive pressure during the second half of expiration rather than inspiration. This is the case for several reasons. First, a small cross-sectional area at end-expiration indicates greater compliance of the pharyngeal walls [26], which, in turn, increases the propensity for obstructive respiratory events [27]. Second, given that the force to dilate the airway is the product of cross-sectional area and pressure [15], expiratory PAP applied prior to the late expiratory reduction in airway size produces a greater dilating force than pressure applied during inspiration (area 4 rather than 1 or 2 in Fig. 2). Third, falling lung volume, which reaches its lowest level at end-expiration, further contributes to increasing pharyngeal collapsibility due to reduced tracheal traction. This has been demonstrated in awake OSA patients [28], but also in the breaths prior to apneas in sleeping OSA participants [22]. During sleep in general, lower end-expiratory lung volume has been directly associated with measures of increased pharyngeal collapsibility [29, 30]; conversely, greater lung volumes yielded lower therapeutic PAP requirements [31]. Real-world examples of this mechanism also come from physiological reductions of lung volume at sleep onset [32] or in supine sleeping position [33], which are typically associated with increased OSA severity [33]. Indeed, decrements in lung volume exert a destabilizing effect on pharyngeal area by reducing caudal traction on the downstream cartilaginous structures, while a larger lung volume tends to stiffen the airway’s walls [34, 35].

The above mechanisms suggest that, with inspiratory pressure relief devices or KPAP treatment, the maintenance of adequate pressure during late expiration likely ensures that the airway is stabilized when inspiration begins, therefore, subsequent inspiratory pressure reductions would not importantly increase collapsibility. However, maintaining some inspiratory positive pressure is arguably important and evidence for this comes from the fact that isolated nasal expiratory PAP does not fully treat OSA [36]. To this end, KPAP never reduces inspiratory pressure below 5 cmH_2_O or more than 5 cmH_2_O below the optimal pressure. Thus, some inspiratory pressure is always provided. In addition, with KPAP, pressure remains reduced at the transition from inspiration to expiration, which means KPAP also includes expiratory pressure relief. However, unlike most prescribed EPRA devices in the US—which restore pressure to therapeutic levels only at the onset of the next inspiration, thus missing the most vulnerable time for pharyngeal collapse—KPAP begins increasing PAP midway through exhalation, returning to full therapeutic pressure well before end-expiration (Fig. 1). This likely increases the probability that sufficient pharyngeal cross-sectional area and end-expiratory lung volume are maintained.

Preliminary in-vivo results [9, 13, 14] support these concepts as there was no loss of efficacy with inspiratory pressure relief devices or KPAP vs. gold-standard PAP treatment. Briefly, the inspiratory pressure relief device was characterized by unchanged residual AHI and therapeutic pressure, lower leak and better adherence vs. PAP therapy [9], while KPAP had similar residual AHI, oxyhemoglobin saturation and reduced leak vs. PAP therapy [13, 14]. In addition, using the inspiratory pressure relief device reduced the occurrence of mouth leak [12] and treatment-emergent central sleep apnea [11]. However, the available findings remain limited by small sample sizes, short treatment durations, lack of real-world validation, and absence of cardiovascular outcomes. Thus, the evidence presented only suggest physiological plausibility and preliminary safety rather than establishing noninferiority or superiority across broad patient populations. Although definitive evidence will ultimately depend on further studies and on independent replication of our results, current physiological understanding seems to suggest that addressing the increased susceptibility to airway collapse with positive pressure in late expiration while reducing inspiratory pressure is at least physiologically sound. Importantly, all the studies on inspiratory pressure relief device and KPAP were conducted in otherwise healthy OSA populations, as these modes are in all likelihood not suitable for patients needing pressure support (e.g., patients with obesity hypoventilation syndrome, neuromuscular disease, advanced chronic obstructive pulmonary disease, etc.), who require increments rather than reductions in inspiratory pressure. Additionally, data stratification to understand whether specific OSA endotypes (which may impact physiological factors such as lung volume or cross-sectional airway area [37]) benefit more than others from reductions in inspiratory pressure has not been conducted to date. Overall, however, these features support the safety and physiological validity of inspiratory pressure relief and KPAP as therapeutic strategies for uncomplicated OSA.

## Data Availability

This manuscript has no associated data.
